# CT Arthrography of the Elbow: What Radiologists Should Know

**DOI:** 10.3390/tomography10030032

**Published:** 2024-03-11

**Authors:** Gianluca Folco, Carmelo Messina, Salvatore Gitto, Stefano Fusco, Francesca Serpi, Andrea Zagarella, Mauro Battista Gallazzi, Paolo Arrigoni, Alberto Aliprandi, Marco Porta, Paolo Vitali, Luca Maria Sconfienza, Domenico Albano

**Affiliations:** 1 Scuola di Specializzazione in Radiodiagnostica, Università degli Studi di Milano, 20122 Milan, Italy; gianluca.folco@unimi.it; 2 IRCCS Istituto Ortopedico Galeazzi, Unità Operativa di Radiologia Diagnostica ed Interventistica, 20157 Milan, Italy; carmelomessina.md@gmail.com (C.M.); sal.gitto@gmail.com (S.G.); stefano.fusco@unimi.it (S.F.); frserpi@gmail.com (F.S.); albanodomenico.md@gmail.com (D.A.); 3 Dipartimento di Scienze Biomediche per la Salute, Università degli Studi di Milano, 20133 Milan, Italy; paolo.vitali@unimi.it; 4 U.O.C. Radiodiagnostica, ASST Centro Specialistico Ortopedico Traumatologico Gaetano Pini-CTO, 20122 Milan, Italy; andrea.zagarella@asst-pini-cto.it (A.Z.); mauro.gallazzi@asst-pini-cto.it (M.B.G.); 5 U.O.C. 1° Clinica Ortopedica, ASST Centro Specialistico Ortopedico Traumatologico Gaetano Pini-CTO, 20122 Milan, Italy; drarrigonielbowlab@gmail.com; 6 Radiology Unit, Zucchi Clinical Institutes Spa, 20900 Monza, Italy; a_aliprandi@yahoo.it (A.A.); porta.marco90@gmail.com (M.P.); 7 Dipartimento di Medicina e Chirurgia, Università degli Studi Milano Bicocca, 20126 Milano, Italy; 8 Unit of Radiology, IRCCS Policlinico San Donato, Via Morandi 30, 20097 San Donato Milanese, Italy; 9 Dipartimento di Scienze Biomediche, Chirurgiche e Odontoiatriche, Università degli Studi di Milano, 20133 Milan, Italy

**Keywords:** CT arthrography, elbow, technique, anatomy, trauma, instability, overuse

## Abstract

Computed tomography (CT) arthrography is a quickly available imaging modality to investigate elbow disorders. Its excellent spatial resolution enables the detection of subtle pathologic changes of intra-articular structures, which makes this technique extremely valuable in a joint with very tiny chondral layers and complex anatomy of articular capsule and ligaments. Radiation exposure has been widely decreased with the novel CT scanners, thereby increasing the indications of this examination. The main applications of CT arthrography of the elbow are the evaluation of capsule, ligaments, and osteochondral lesions in both the settings of acute trauma, degenerative changes, and chronic injury due to repeated microtrauma and overuse. In this review, we discuss the normal anatomic findings, technical tips for injection and image acquisition, and pathologic findings that can be encountered in CT arthrography of the elbow, shedding light on its role in the diagnosis and management of different orthopedic conditions. We aspire to offer a roadmap for the integration of elbow CT arthrography into routine clinical practice, fostering improved patient outcomes and a deeper understanding of elbow pathologies.

## 1. Introduction

In recent years, diagnostic imaging has undergone remarkable advancements, transforming the landscape of medical practice and significantly enhancing the accuracy and precision of clinical diagnoses [1]. Among the myriad techniques that have emerged, computed tomography (CT) arthrography has gained substantial attention within the field of musculoskeletal radiology [2,3]. CT arthrography is now a well-established imaging technique, which made its initial appearance in radiological literature between the late 1970s and the early 1980s to assess the integrity of intra-articular structures, such as the cruciate ligaments of the knee [4]. When iodinated contrast medium is injected into the joint, it enhances the visibility of intra-articular structures, such as cartilage and ligaments, as their low attenuation results in high contrast with the intra-articular radiopaque injectate [5]. Hence, this imaging modality combines the power and high spatial resolution of CT with the diagnostic capabilities of arthrography, providing clinicians with a detailed visualization of joint structures. As articular disorders continue to pose challenges in diagnosis and management, CT arthrography emerges as a pivotal tool, offering unparalleled insights into the intricacies of joint pathology [6].

While magnetic resonance (MR) and MR arthrography are widely regarded as the reference standard for nonoperative elbow imaging owing to high sensitivity and specificity [7,8,9,10], CT arthrography represents a valuable complementary diagnostic tool for addressing a variety of clinical queries [11]. The use of multichannel detector arrays, the availability of submillimeter-thick slices, and new methods to reduce metal-related beam hardening artifacts, has enabled the examination of joints with remarkable precision, particularly by allowing isotropic multiplanar imaging reformatting with excellent spatial resolution. CT arthrography is particularly indicated in cases where MR or MR arthrography have shown to be ineffective, unavailable, or when dealing with patients who are obese, severely claustrophobic, with MR-incompatible implanted medical devices, or bearing metal implants that may impair image quality [12]. Time is another key advantage of CT arthrography when compared to MR arthrography: while a CT arthrography scan requires mere seconds of still posture, a MR arthrography scan needs the patient to remain completely motionless for several minutes, often within a confined gantry with frequently an uncomfortable arm position [13]. The protracted immobility can be challenging for certain patients, especially those experiencing severe pain. This may be especially relevant when imaging the elbow, as it may require more difficult positioning compared to other articulations. In evaluating the elbow, CT arthrography may be employed for the assessment of joint surfaces, loose bodies, and ligamentous structures of the elbow. In the setting of either acute traumatic events, such as dislocation, or chronic overuse due to repetitive microtrauma, identification of injury entity and pattern may help the clinician guide patient management [14].

This comprehensive review aims to delve into the evolution and technical principles of CT arthrography providing an overview of the technique, while showcasing the practical applications of CT arthrography in various clinical scenarios, shedding light on its role in the diagnosis and management of different orthopedic conditions ranging from the evaluation of traumatic injuries and degenerative joint diseases to the assessment of soft tissue abnormalities. We aspire to offer a roadmap for the integration of elbow CT arthrography into routine clinical practice, fostering improved patient outcomes and a deeper understanding of elbow pathologies.

## 2. Technique

The success of elbow CT arthrography lies in the meticulous execution of the technique, involving the careful administration of contrast agent and the patient positioning. Elbow CT arthrography can be conducted in one of two modalities: either as a single-contrast examination, involving the use of air or iodinated contrast, or as a double-contrast examination, which entails the injection of a small dose of iodinated contrast followed by the introduction of air. The choice between these techniques is generally guided by personal preference and experience. A single-center study has suggested the use of dimeric contrast agents as an alternative to conventional monomeric contrast agents to enhance the retention of iodinated contrast media within the joint [15]. At the authors’ institutions, a single-contrast technique is preferred: 5–7 mL of injectate constituted of a conventional monomeric iodinated contrast agent (e.g., iopamidol at 33 mg/mL) diluted to a 60% concentration with saline is employed. Doses slightly higher than 5 mL—the average capacity of the elbow joint [16]—should be considered in patients with a large habitus.

Regarding the intra-articular injection technique, modern practice increasingly favors the use of ultrasound (US) guidance over fluoroscopy guidance, a preference held by many specialists [17,18,19]. US stands out as a cost-effective, safe, highly efficient, and radiation-free modality, ideal for conducting various interventional procedures, including intra-articular injections [18,19,20]. Notably, US guidance provides real-time visualization of the needle, affording a clear view of its tip as it enters the joint space. It also allows for close monitoring of the injection of contrast as it distends the articular recess [21]. Furthermore, US guidance permits intra-procedural visualization of neurovascular structures. In addition, a pre-procedural US evaluation carried out with a linear, high-frequency transducer and encompassing both static and dynamic scanning, might serve as a complementary diagnostic tool, allowing for a preliminary evaluation of periarticular soft tissues, such as tendons and ligaments.

**Figure 1:**
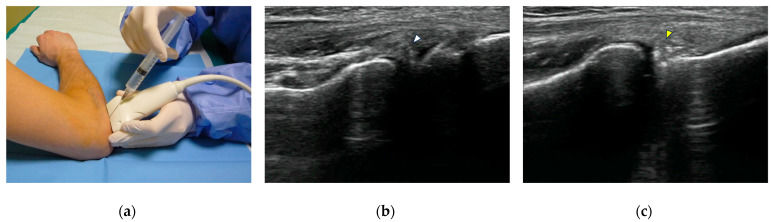
US-guided intra-articular injection. (**a**) The injecting needle is inserted at the level of the radio-humeral joint, using an out-of-plane approach, with the transducer positioned along the long axis of the common extensor tendon; (**b**) US guidance allows monitoring the intra-articular placement of the needle tip (white arrowhead) and demonstrates the correct outflow of injectate; (**c**) as the injection is complete and the needle is withdrawn, hyperechoic intra-articular contrast medium is visualized in the radio-humeral recess (yellow arrowhead).

When preparing for elbow arthrography, a small, 1.5-inch (3.8 cm) 25G needle is inserted either in the radio-humeral compartment, with the patient exposing the lateral elbow by sitting down (Figure 1) or lying supine with the arm behind the back, or in the olecranon fossa with a posterior transtriceps approach [16]. The injecting needle can be positioned in an in-plane modality to allow for tracking of its trajectory from the access point to the intended target site, or in an out-of-plane modality, depending on operator preference [22]. To mitigate the discomfort associated with intra-articular puncture, a local anesthetic agent, typically 2–3 mL of lidocaine hydrochloride, may be administered. If excessive resistance is encountered during injection, and contrast fails to flow into the articular recess as anticipated, the needle should be slightly withdrawn and repositioned. As a rule of thumb, the CT arthrography examination should be performed as soon as possible after the injection to avoid compromising image quality, as intra-articular iodinated contrast will begin to disperse rather quickly: an in vivo study has shown that its half-life within the joint space can range from 30 to 60 min.

Elevating the arm, rather than having it positioned adjacent to the torso, results in a substantial reduction in the absorbed radiation dose, with reported mean effective doses of 0.21 (±0.11) millisievert [23]. The patient is generally positioned prone on the CT scan table with the elbow over head with a 45° flexion. Before scanning, scout images are obtained, which may help identifying loose bodies, metal hardware, and calcific densities such as chondrocalcinosis. These radiographs from the arthrogram serve to validate the findings observed in the subsequent CT arthrography study. The CT arthrography examination is then conducted by obtaining overlapping submillimeter isotropic images, typically 0.5 to 0.8 mm thick. The field of view should be precisely targeted to maximize the detail of the joint, including the distal part of the humerus, starting from above the epicondyles, and the proximal forearm, to the radial tuberosity. A medium-sharp algorithm is employed for reconstructing the images. Upon concluding the examination, multiplanar reformatted images are generated at the CT console to observe the joint surfaces in anatomical planes. Soft and bone kernel reconstructions, generally with 1 mm slice thickness, should be produced on the axial, coronal, and sagittal planes. The total duration of a CT arthrography procedure, from US injection to CT arthrography acquisition, is typically around 15–20 min.

## 3. Anatomy

The elbow joint is a complex articulation that serves as a pivotal link between the upper arm and forearm, facilitating a wide range of movements essential for daily activities. It is a synovial hinge joint, primarily responsible for flexion and extension, as well as some rotational movements. The anatomy of the elbow joint involves the coordination of three main bones: the humerus, the radius, and the ulna. Indeed, the elbow consists of three joints enclosed in a single synovial capsule: the radio-humeral, the ulno-humeral, and the radio-ulnar joints [24,25]. Even though CT arthrography allows an accurate evaluation of articular cartilage, especially at the radio-humeral joint, the elbow is a technically demanding joint with regard to cartilage imaging: it displays a chondral thickness of 1–2 mm or less, in contrast to thickness values of 5 mm or more in the knee [26,27]. As such, the use of elbow CT arthrography should only be recommended with the availability of modern submillimeter, multi-slice CT equipment [27].

**Figure 2:**
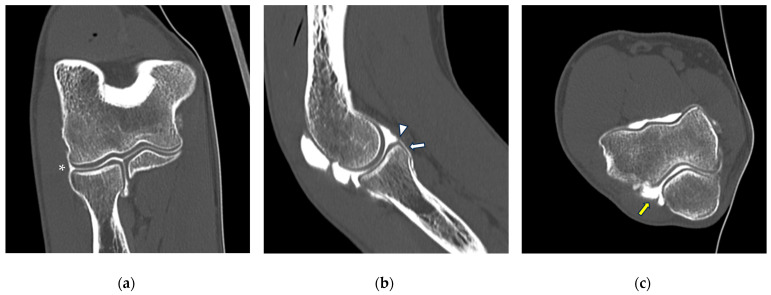
CT arthrography of the elbow—normal anatomy. (**a**) Coronal image shows a regular chondral lining of the radio-humeral, ulno-humeral, and radio-ulnar joints, with no evidence of focal chondral lesions. The articular capsule is not pathologically distended and there is only a physiological amount of contrast in the lateral radio-humeral recess (asterisk), indicating the presence of an intact radial collateral ligament; (**b**) sagittal image shows an undisplaced annular ligament (white arrowhead), as well as a regular chondral lining of the radial head side and a physiological sliver of contrast in the annular recess (white arrow); (**c**) axial image shows a physiological amount of fluid in the posterior recess of the elbow (yellow arrow), with no evidence of loose bodies, as well as regular chondral lining of the humerus and olecranon.

The joint is encapsulated by an articular capsule, a structure that plays a pivotal role in stabilizing the articulation and maintaining joint integrity. The stability of the elbow joint is further fortified by a network of ligaments, each with distinct functions. Indeed, ligamentous structures of the elbow originate as focal thickenings of the joint capsule and are divided into ulnar and radial collateral ligament complexes. As an injury of these elbow stabilizers may lead to elbow instability [28], elbow CT arthrography can provide useful information regarding their integrity. The medial collateral ligament (MCL) complex is made by anterior, posterior, and transverse bundles and acts as the main stabilizer against valgus and extension stress [29], with medial elbow pain being a common symptom in overhead-throwing athletes: in these patients, the medial compartment is subjected to chronic tensile forces that result in inflammation, micro-tearing, and eventually ligament disruption [30]. The lateral collateral ligament (LCL) complex is composed by the radial collateral ligament (RCL), annular ligament, and lateral ulnar collateral ligament (LUCL), stabilizing the elbow against varus and external rotational stress. The annular ligament, encircling the head of the radius, maintains the proper relationship between the radius and ulna during rotational movements. Injuries are more common in the setting of an acute event, such as elbow dislocation, with overuse-related conditions being somewhat more uncommon than in the medial compartment [10]. A comprehensive understanding of the capsule and ligamentous structures is imperative for radiologists, as it forms the foundation for accurate interpretation of CT arthrography, aiding in the diagnosis of traumatic injuries, instability, and pathological conditions affecting the elbow joint. An example of normal CT arthrography anatomy of the elbow is shown in Figure 2.

## 4. Osteochondral Pathology

Elbow cartilage is a critical component that plays a pivotal role in maintaining the integrity and functionality of the elbow joint. Composed predominantly of hyaline cartilage, this resilient and avascular tissue covers the articular surfaces of the distal humerus, proximal ulna, and radial head within the joint [27]. The assessment of elbow cartilage has been significantly enhanced by the capabilities of CT arthrography, which enables high-resolution, multiplanar visualization of the articular surfaces, allowing the detection of subtle changes in cartilage thickness, integrity, and contour, contributing to a detailed understanding of pathology [31]. As a matter of fact, traumatic and degenerative pathology affecting cartilage and subchondral bone can be investigated through CT arthrography [32].

**Figure 3:**
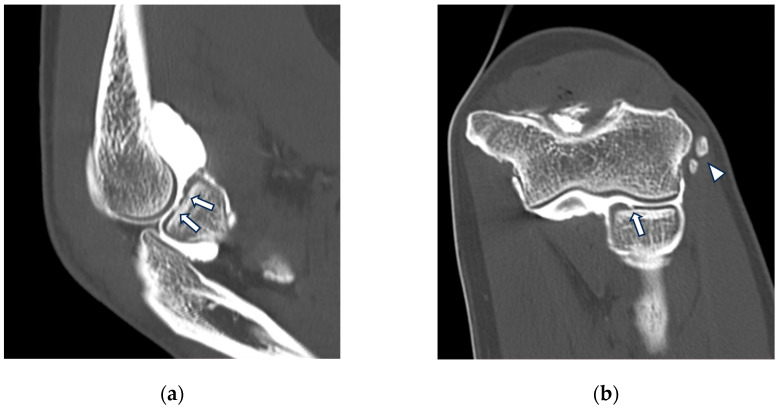
CT arthrography of a patient with chronic elbow instability. (**a**) Sagittal image shows pitting and fissuring of radial head cartilage, involving >50% of its thickness (grade III) (white arrows); (**b**) coronal reformat of the same patient shows a focal full-thickness cartilage defect of the anteromedial radial head (grade IV) (white arrow). In the same image, mature calcifications can be seen at the insertion of the common extensor tendon (white arrowhead).

A thorough cadaveric study with single-contrast technique has shown modern CT arthrography and MR arthrography to have similar sensitivity (87% and 85%, respectively) and specificity (94% and 95%, respectively) in the detection of chondral injuries of the elbow joint [33]. The same study also suggested that CT arthrography is likely to outperform MR arthrography under in vivo conditions, due to the higher susceptibility of the latter to motion artefacts. Even though diagnosis of early stage chondromalacia has been shown to be rather limited at both MR arthrography and CT arthrography, the same study displayed excellent detection rates for grade III and IV cartilage lesions of the capitellum and radial head, with CT arthrography displaying a slightly higher sensitivity. As the radio-humeral compartment represents the most frequent site of elbow chondromalacia [34], these results support the use of elbow CT arthrography for routine evaluation of articular surface degenerative changes (Figure 3). Based on arthroscopic findings, the modified Outerbridge classification is a widely used grading system of degenerative articular cartilage lesions, encompassing: grade I (initial swelling), grade II (superficial fraying and fissuring), grade III (partial-thickness chondral loss), and grade IV (full-thickness chondral loss with exposed subchondral bone) lesions [35].

**Figure 4:**
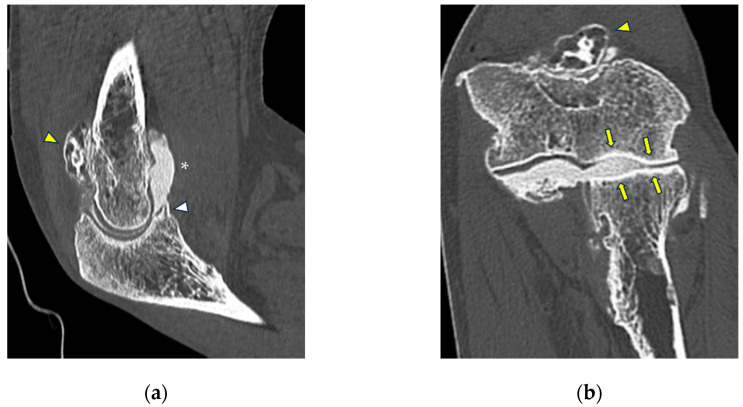
CT-arthrography of a patient with rheumatoid arthritis. (**a**) Sagittal image shows pathological widening of the anterior humero-ulnar recess (asterisk), osteophytosis of the coronoid process (white arrowhead) and a distended posterior recess containing multiple non-calcific loose bodies compatible with secondary synovial chondromatosis (yellow arrowheads); (**b**) coronal image shows abnormal morphology and complete chondral erosion of both the radial head dish and lateral humeral condyle (yellow arrows).

Loose bodies may result from osteochondritis dissecans (OCD), osteoarthritis, synovial chondromatosis (Figure 4), and osteochondral fractures. Even though elbow CT arthrography can detect loose bodies as small as 3 mm in diameter, plain radiography has been shown to have comparable sensitivity (84%) and specificity (71%). On the other hand, while plain radiography is a readily available first-level technique, it is often inaccurate: primary chondral lesions are not seen, it is difficult to distinguish between osteophytes and loose bodies and to determine whether the loose body is intra-articular [36]. The same study has shown CT arthrography to be more accurate than MR arthrography in determining loose body location, whether intra- or extra-articular [36]. OCD of the elbow typically affects the central and anterolateral aspect of the capitellum, most commonly related to repetitive valgus impaction injury of the radial head [37]. The development of OCD is thought to arise from a compromised vascular supply to the capitellum, leading to the fragmentation of osteochondral tissue and, ultimately, instability [38]. Instability of the osteochondral fragment can be assessed at CT arthrography by the visualization of a surrounding rim of intra-articular contrast [39]. Arthrographic examinations have a unique role to evaluate osteochondral injuries, being essential in determining the stability of such lesions. This determination is pivotal in deciding whether surgical intervention is necessary. OCD should be differentiated from Panner’s disease, a benign osteochondrosis of the capitellum that shows involvement of the whole epiphysis, does not result in loose body formation, and typically shows a subchondral vacuum phenomenon characterized by a crescent-shaped rim with air attenuation [40].

When evaluating the elbow cartilage, one must consider that there can be a highly variable area of the ulnar trochlear fossa which may appear bare, yielding an intrinsic risk of misdiagnosis of cartilage ulceration. One study observed that this bare spot is located where the ossification centers of the proximal ulna are fused, approximately 15.8 mm from the tip of the olecranon and 13.8 mm from the coronoid process [41].

Foreign bodies may also represent an issue in articular imaging, especially when wooden, as this material is not as easily detectable from X-ray, which is usually the main first level technique in this setting. While US may indeed be a reasonable choice, in case of prior trauma or other indications, CT and CT arthrography imaging also displayed a satisfactory ability in identifying wooden foreign bodies [42].

## 5. Capsule and Ligaments

The main causes of damage to the capsule and ligaments of the elbow joint are represented by acute trauma and chronic injury due to repeated microtrauma and overuse.

### 5.1. Acute Trauma

It is important to note that after an acute traumatic event the joint capsule can be distended by articular effusion, or the capsule may be damaged. As such, arthrographic imaging is usually discouraged in the acute setting immediately following a traumatic event [43]. Indeed, arthrographic imaging is usually performed to evaluate extra- and intra-articular structures in case of residual deficits, after the acute post-traumatic stage is past. Further, when a great amount of joint effusion is present, MR or CT can be performed without intraarticular contrast injection, with a similar arthrographic effect.

**Figure 5:**
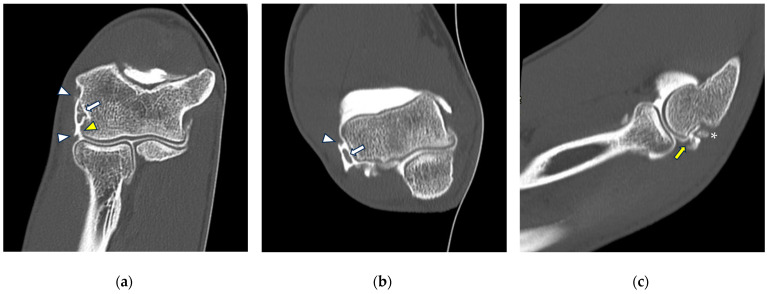
CT arthrography of a patient with sequelae of traumatic elbow dislocation. (**a**,**b**) Coronal and axial images showing LCL laxity with subtle foci of contrast medium permeation due to ligament delamination (white arrowheads) and a loose body within a pathologically distended lateral recess (white arrow), as well as cartilage thinning and fraying of the lateral aspect of the capitulum humeri (yellow arrowhead). Post-traumatic deformity of the radial head can also be seen; (**c**) sagittal reformat shows a loose bony fragment posterior to the capitulum humeri (asterisk) and posterior synovial thickening (yellow arrow).

Acute trauma to the elbow most often leads to ligament tears, more commonly the LCL—in case of varus stress—and less commonly the MCL, in case of valgus stress [44]. This may be represented, for instance, by a fall on outstretched arm for the MCL. Full-thickness tears of the MCL or LCL can be identified on CT arthrography as discontinuity of the ligament with extravasation of intra-articular contrast medium into peri-articular soft tissues (Figure 5) [31]. While it is also important to detect partial ligament tears, as they may warrant surgical reconstruction in athletes, their diagnosis can be more difficult, especially on MR imaging [45]. Conversely, the higher spatial resolution of CT arthrography may yield a higher sensitivity, thus allowing the detection of focal ligament thinning and subtle permeation of the ligament by intra-articular contrast medium filling the tear and extending into the ligament due to fiber delamination [11]. The typical finding of a distal partial tear of the MCL is the so-called “T sign”, related to contrast permeation into the defect and extending distally between the MCL and the proximal aspect of the ulna [25]. Indeed, the anterior bundle of the MCL in normal conditions attaches about 3 mm distal to the proximal profile of the sublime tubercle; hence, a partial tear might be considered when contrast extends more than 3 mm distally [46]. In the setting of acute trauma, there are often concomitant injuries to adjacent structures, such as capsular disruption, fractures of the coronoid process, delamination of articular cartilage, and contusions [46]. The most reliable indication of traumatic disruption to the MCL is the finding of contrast extending medially beyond the joint line [46]. In the context of trauma, the tear’s specific location (proximal or distal) becomes less crucial, and such tears seldom need reconstruction.

Concerning the LCL complex, the LUCL is the lateral ligament most commonly injured in cases of traumatic elbow subluxation or dislocation [47]. The identification of focal thickening or laxity in ligaments should induce prompt consideration of a potential partial tear. Full thickness tears may manifest as distinct disruptions with the extravasation of contrast through the defect [47]. Partial or complete disruption of the LCL complex can lead to posterolateral rotatory instability (PLRI), usually following traumatic dislocation [48]. PLRI may be divided into three stages according to the level of soft tissue damage, extending from the lateral to the medial compartment. Stage 1 PLRI is represented by posterolateral subluxation of the ulna, with lateral ulnar collateral ligament damage. Stage 2 PLRI presents with an incompletely luxated elbow, with the coronoid process under the trochlea, and disruption to the lateral ulnar and radial collateral ligament and capsule. Stage 3 PLRI adds progressive disruption of the MCL, which can be partial or complete [43,49].

### 5.2. Chronic Injuries and Overuse

**Figure 6:**
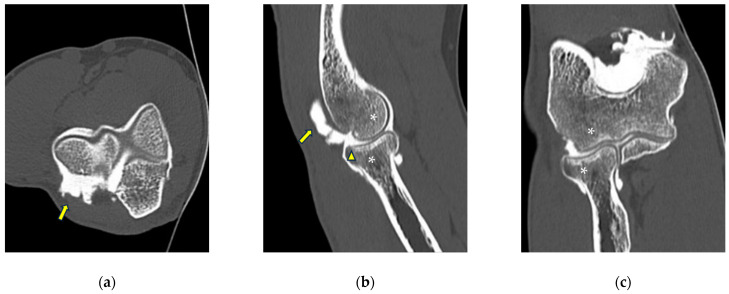
CT-arthrography of a professional motocross rider with chronic elbow pain due to repeated microtraumatism. (**a**,**b**) Axial and sagittal images show pathological widening of the postero-lateral recess (yellow arrows); (**b**,**c**) sagittal and coronal images show articular asymmetry of the humero-radial joint (asterisks), diffuse cartilage fraying and a full-thickness chondral defect (grade IV) of the posterior aspect of the radial head dish (yellow arrowhead).

MCL injuries are typically caused by overuse [44]. Chronic injuries to the MCL may lead to medial elbow instability [50], which most often occurs in athletes who participate in overhead throwing sports such as baseball, javelin throwing, volleyball, golf, polo, and football. As stated previously, the main types of ligaments lesions that can be visualized at CT arthrography are represented by thickening or full-thickness or partial tears, which, however, in this setting are usually accompanied by other chronic local changes, such as the formation of osteophytes, presence of loose bodies or synovial thickening (Figure 6) [51]. The medial compartment may also present with posteromedial elbow impingement, which may in turn be associated with elbow instability due to MCL insufficiency [52]. Posteromedial elbow impingement is considered as a feature of valgus extension overload syndrome, presenting with pain, swelling, chondral injuries located into the posterior trochlea and the anterior and medial olecranon, postero-medial osteophytes, and loose bodies [52]. Imaging is essential for treatment decision-making, given that MCL tears would need surgical reconstruction after removal of osteophytes and loose bodies [53].

**Figure 7:**
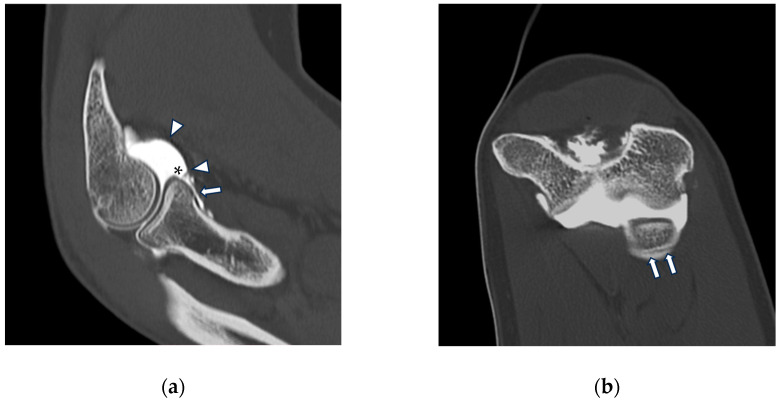
CT arthrography of a patient with overuse-related lateral elbow pain. (**a**) Sagittal image shows pathological widening of the radio-humeral and radio-annular recesses (white arrowheads) due to ligamentous laxity—in particular, the annular ligament (white arrow) is displaced distally to the proximal half of the radial head side, which also shows anterior cartilage thinning and synovial thickening (asterisk); (**b**) the corresponding coronal reformat may aid in precise localization of the proximal extremity of the annular ligament, which can be visualized as a hypodense band anterior to the radial head (white arrows); in this instance, it shows marked distal displacement.

**Figure 8:**
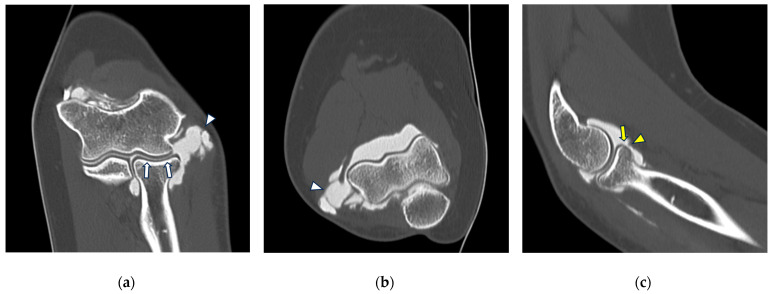
CT arthrography of a patient with lateral epicondylitis subjected to multiple corticosteroid injections. (**a**,**b**) Coronal and axial images show extravasation of intra-articular contrast into lateral periarticular soft tissues through a large full-thickness tear of both the radial collateral ligament and the proximal common extensor tendon (white arrowheads). Diffuse thinning of radial head dish cartilage is also displayed (white arrows); (**c**) sagittal image also shows distal displacement of the annular ligament (yellow arrowhead) and cartilage fraying of the radial head side (yellow arrow).

Overuse-related lateral elbow pain, often diagnosed as lateral epicondylitis, may be often associated with a condition of elbow instability termed symptomatic minor instability of the lateral elbow (SMILE), which has been shown to present with several intra-articular findings detectable at CT arthrography, such as radiocapitellar chondromalacia and pathological laxity of the annular ligament (Figure 7) [54]. Extreme cases of concomitant LCL and common extensor tendon full-thickness rupture can also sometimes be seen as sequelae of repeated corticosteroid injections performed without US guidance for the treatment of lateral epicondylitis [55], with extravasation of intra-articular contrast agent in peritendinous soft tissues (Figure 8).

## 6. Future Insights and Conclusions

While MR arthrography is the most common choice when performing arthrographic imaging of the elbow, CT arthrography has gained ground in more recent years, in view of the notable technological advancements of CT that led to even higher spatial resolution and ease of use. In this scenario, while the primary drawback of CT arthrography is the exposure to ionizing radiations, new technologies and protocols led to the reduction of radiation exposure in joint imaging. For instance, despite their higher costs, new photon-counting detector CT scanners have been praised for being capable of acquiring images with superior anatomical detail, with image thickness as low as 0.2 mm, and for yielding substantial reductions in radiation dose, with a decrease in effective dose reported as low as 65% for bone imaging [56,57]. Other studies proposed a C-arm flat-panel CT arthrography framework, which may allow for higher spatial resolution for articular imaging. A proof-of-concept study in this regard tested this technique on human cadaveric elbows to determine the optimal intra-articular iodine concentration in this setting, observing that a concentration of 45 mgI/mL allows for superior contrast-to-noise ratio than conventional CT arthrography [58]. As technology continues to evolve, future developments in CT arthrography may further refine our diagnostic capabilities and contribute to improved patient outcomes. Hence, CT arthrography imaging of the elbow may provide a valuable alternative to MR arthrography in those patients who cannot undergo the latter due to contraindications or lack of access, also adding the benefit of an increased spatial resolution which can help identify more subtle conditions.

In conclusion, this review article has provided a comprehensive exploration of the utility and nuances of CT arthrography in the assessment of the elbow joint, offering valuable insights for radiologists engaging in musculoskeletal imaging. The anatomical intricacies of the elbow joint, including the articular capsule and ligamentous structures, have been highlighted as critical components for radiologists to interpret and analyze during CT arthrography examinations. The ability of CT arthrography to delineate soft tissue structures and visualize subtle abnormalities, particularly in cases of ligamentous injuries and subtle articular pathology, positions it as an invaluable tool in the diagnostic armamentarium for elbow joint assessments. Furthermore, the review has emphasized the expanding clinical applications of CT arthrography, ranging from traumatic injuries to degenerative conditions, thereby reaffirming its significance in comprehensive musculoskeletal care.

## Data Availability

No new data were created or analyzed in this study. Data sharing is not applicable to this article.
